# Supplementary material for: Warming underpins community turnover in temperate freshwater and terrestrial communities
Source: Nat Commun. 2024 Mar 1;15:1921. doi: 10.1038/s41467-024-46282-z (PMC10907361; doi:10.1038/s41467-024-46282-z)
Supplement: Supplementary file 1 — Supplementary Information [file 41467_2024_46282_MOESM1_ESM.pdf]

Supplementary Files.

Warming-related community turnover is weaker in freshwater than in terrestrial ecosystems

Imran Khaliq[1,2,3,4], Christian Rixen[2,3], Florian Zellweger[5], Catherine Graham[5], Martin M. Gossner[5,6], Ian R. McFadden[5,6], Laura Antão[7], Jakob Brodersen[8], Shyamolina Ghosh[5], Francesco Pomati[1], Ole Seehausen[8], Tobias Roth[9,10], Thomas Sattler[11], Sarah R. Supp[12], Maria Riaz[13,14], Niklaus Zimmermann[5,6], Blake Matthews[8], Anita Narwani[1]

Supplementary Information:

Fig. S1| Spatial distribution and temperature changes observed over the duration of community time-series. In panels **a** and **b,** coloured dots show rates of significant temperature change over the duration of the community time-series for the terrestrial and freshwater realms, respectively, where red indicates warming and blue indicates cooling, with more intense shades indicating greater rates of temperature change. Black dots represent sites where rates of temperature change were not significantly different from zero. **c**) Density distributions of rates of temperature change observed at the sites by realm in panels **a** and **b**. The inset in panel **c** shows the mean temperature change in the freshwater and the terrestrial realms.

Fig. S2| Thermophilisation rates across realms and taxonomic groups. Mean thermophilisation rates by taxonomic group, showing the change in the community temperature index (CTI) over time, estimated for each community and averaged across communities within each taxonomic group (numbers in parentheses indicate the number of time-series for each taxonomic group). Rates are significantly different from zero for all groups. Error bars represent the 95% confidence intervals of the mean. Silhouettes were created with BioRender.com

Fig. S3 | Interactive effects of temperature change and realm on thermophilisation. The model included temperature change and realm as fixed factors with an interaction term, with study ID was nested within taxonomic group as random factors (Table S1). The solid blue line represents marginal effects of temperature change for the freshwater realm, and the dashed green line represents marginal effects of temperature change for the terrestrial realm. The thinner exterior curves represent standard errors.

Fig. S4| Predictors of thermophilisation across realms and taxonomic groups (excluding the tropical and polar communities). The plotted points show mean effect sizes of temperature change, mean community body size, mean thermal niche breadth, baseline mean annual temperature, time-series length and species richness on the rates of thermophilisation. Panels **a** and **e** are for all the taxonomic groups plotted for the terrestrial and the freshwater realms, respectively; panels **b-d** and **f-h** are plotted for particular taxonomic groups: plants, terrestrial insects, and birds (**b**-**d**), and zooplankton, aquatic insects, and fish (**f**-**h**). Please note that the x-axis range differs among the panels, though the dashed line for zero is the same on all plots. For each realm, effect sizes were calculated after removing outliers that lie beyond two standard deviations from the mean, and after accounting for the effects of taxonomic group (random factor), study id (random factor) and spatial autocorrelation. We also estimated the interaction effects of body size, thermal niche breadth, baseline temperature and temperature change. Effect sizes with grey circles are not significantly different from zero based upon error bars represent 95% confidence intervals are overlapping zero. Silhouettes were created with BioRender.com

Fig. S5| Predictors of thermophilisation across realms and taxonomic groups (including mammals and phytoplankton). The plotted points show mean effect sizes of temperature change, mean community body size, mean thermal niche breadth, baseline mean annual temperature, time-series length and species richness on the rates of thermophilisation. Panels

a and **c** are for all the taxonomic groups plotted for the terrestrial and the freshwater realms, respectively; panels **b** and **d** are plotted for mammals and phytoplankton. Please note that the x-axis range differs among the panels, though the dashed line for zero is the same on all plots. For each realm, effect sizes were calculated after removing outliers that lie beyond two standard deviations from the mean, and after accounting for the effects of taxonomic group (random factor), study id (random factor) and spatial autocorrelation. We also estimated the interaction effects of body size, thermal niche breadth, baseline temperature and temperature change. Effect sizes with grey circles are not significantly different from zero based upon error bars represent 95% confidence intervals are overlapping zero. Silhouettes were created with BioRender.com

Fig. S6| Contributions of immigration and extirpation to thermophilisation. Difference in the mean thermal affinities of species that immigrated (added), that persisted or that were extirpated (lost) from individual communities. The individual points represent pairwise differences in the mean thermal affinities between these groups (i.e. added, persisted or lost species) for each community. Asterisks indicate whether differences are significantly more frequently above or below zero than expected based on a 0.5 probability (binomial-test); this is indicated by the position of the asterisks above or below zero on the y-axis. * indicates $P < 0.05$, ** indicates $P < 0.005$, ***indicates $P < 0.001$. Silhouettes were created with BioRender.com

Fig. S7| Species temperature index (STI) and mean annual temperatures for each community. STI was calculated as the average mean annual temperature across each species distributional range. The solid black line indicates a 1:1 relationship between STI and average mean annual temperature. Data points above the black line indicate that STI is higher than the average mean annual temperature at a local site and vice versa.

Fig. S8| Relationship between two measures of CTI. Relationship between the community temperature index (CTI) weighted by the abundance of species (y-axis) and the CTI based upon species occurrence data (x-axis) show highly correlated values.

Fig. S9| Plant CTI estimated from distributional data and from Ellenberg's T indicative values. For plants CTI was calculated using species level distributional data downloaded from GBIF and classification taken from the Ellenberg's[49] temperature indicative values. CTI calculated based upon two measures yielded highly correlated values.

Fig. S10| STIs estimated using shape files of species' ranges against species STIs estimated from GBIF distributional data. We downloaded range maps from the BirdLife dataset for birds and for plants we used the thermal preference data from reference[8] based upon range maps. We overlayed the mean annual temperature layer on the range maps and calculated the species thermal preference for each species as average mean annual temperatures across the whole geographic distribution separately. We also calculated the average mean annual temperature using GBIF data. The correlations indicate that the two measures are comparable and yield highly correlated values.

Fig. S11| STI estimated using distributional data before year Pre-1990, 1990-1991, 2001-2010, and post-2010 periods. For all taxonomic groups, STIs were calculated by sub-setting the GBIF occurrence data using above time bins. STI values for both the realms across all measures are highly correlated. Occurrence records for mammals and phytoplankton are not included.

Fig. S12| CTI plotted as a function of the local sites' mean annual temperature. Site-level mean CTI and site-level mean annual temperatures were calculated across all years for each site. There is a positive relationship observed for all the taxa. All relationships are statistically significant at $\alpha=0.05$, except for phytoplankton.

Fig. S13| Relationship of CTI based upon all the species present in community and CTI based upon species with more than 5 occurrence records. CTI values on y-axis were calculated while excluding all species for which less than five occurrence information was available. On the x-axis, we included all the species present in a community.

Fig. S14| Correlation matrices of predictor variables for aquatic and terrestrial realms. Colours range from white (negative correlation) to dark blue (positive correlation).

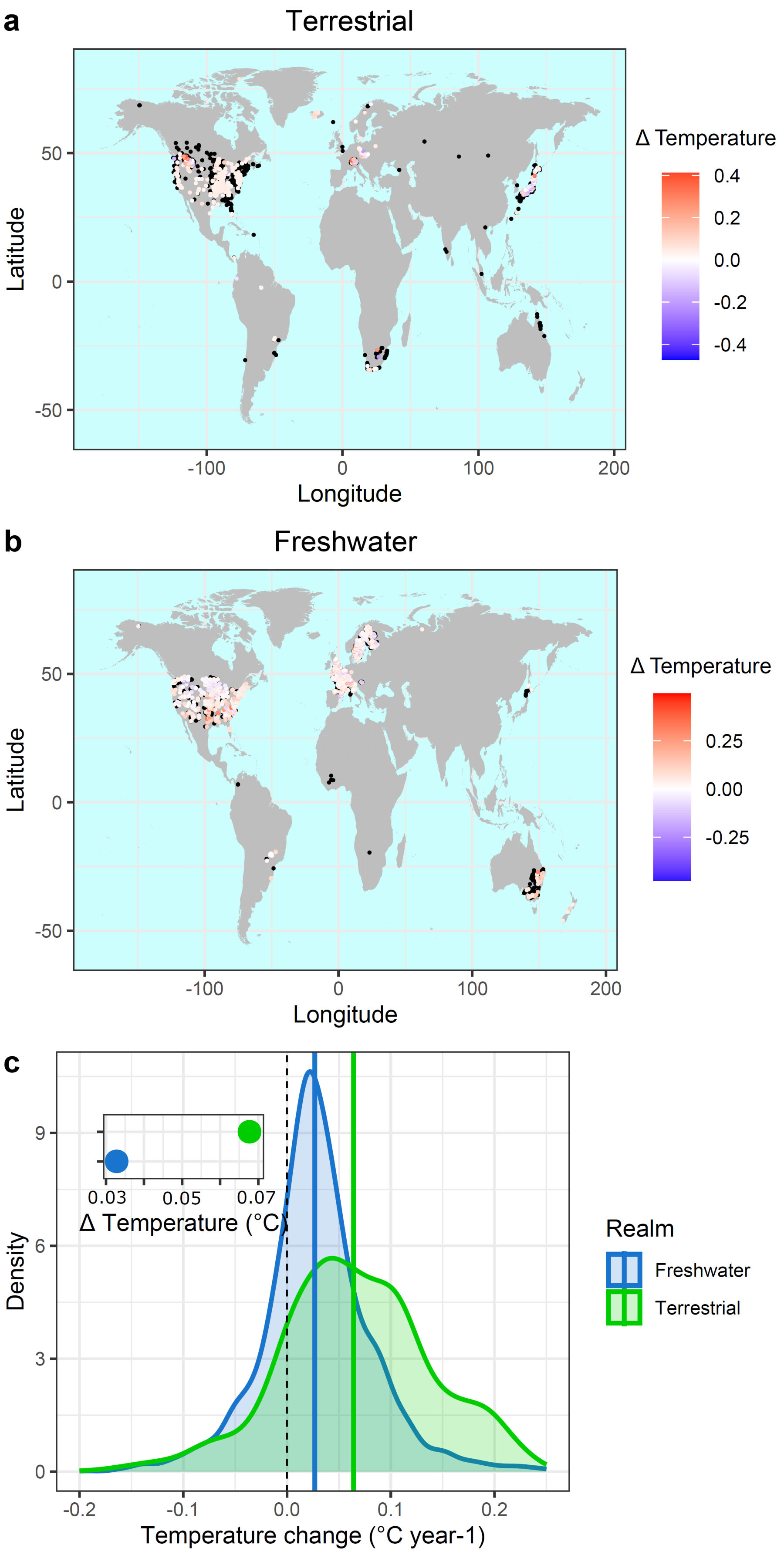
a
Terrestrial
Latitude
Longitude
Δ Temperature
0.4
0.2
0.0
-0.2
-0.4
b
Freshwater
Latitude
Longitude
Δ Temperature
0.25
0.00
-0.25
c
Δ Temperature (°C)
Density
Temperature change (°C year-1)
Realm
Freshwater
Terrestrial

121

Fig. S1| Spatial distribution and temperature changes observed over the duration of community time-series. In panels **a** and **b,** coloured dots show rates of significant temperature change over the duration of the community time-series for the terrestrial and freshwater realms, respectively, where red indicates warming and blue indicates cooling, with more intense shades indicating greater rates of temperature change. Black dots represent sites where rates of temperature change were not significantly different from zero. **c**) Density distributions of rates of temperature change observed across the sites by realm. The inset in panel **c** shows the mean temperature change in the freshwater and the terrestrial realms.

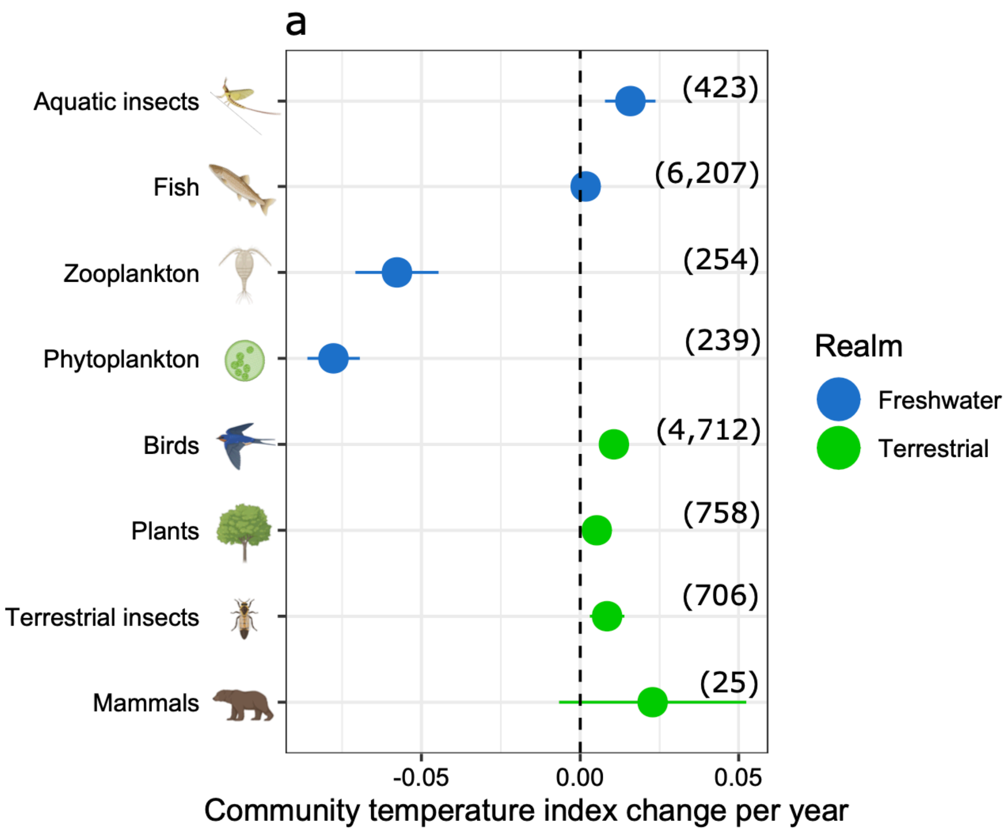

Fig. S2| Thermophilisation rates across realms and taxonomic groups. Mean thermophilisation rates by taxonomic group, showing the change in the community temperature index (CTI) over time, estimated for each community and averaged across communities within each taxonomic group (numbers in parentheses indicate the number of time-series for each taxonomic group). Rates are significantly different from zero for all groups. Error bars represent the 95% confidence intervals of the mean. Silhouettes were created with BioRender.com

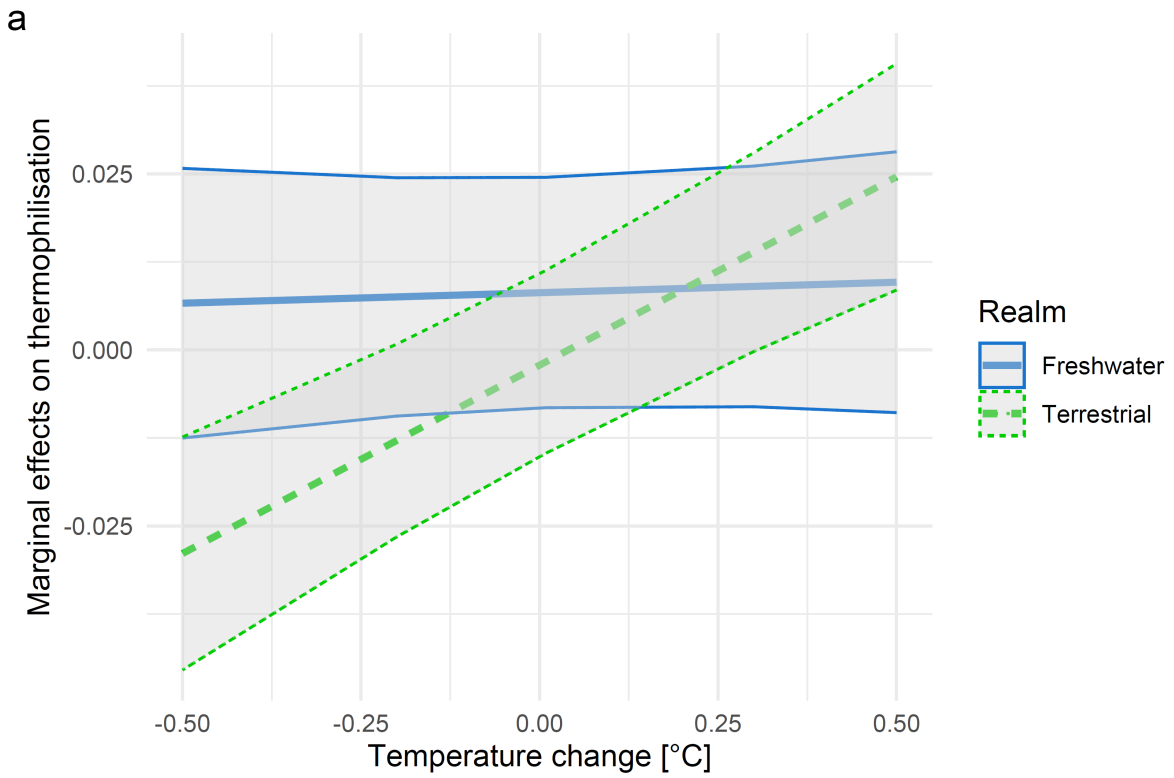

Fig. S3| Interactive effects of temperature change and realm on thermophilisation. The model included temperature change and realm as fixed factors with an interaction term, with study ID was nested within taxonomic group as random factors (Table S1). The solid blue line represents marginal effects of temperature change for the freshwater realm, and the dashed green line represents marginal effects of temperature change for the terrestrial realm. The thinner exterior curves represent standard errors.

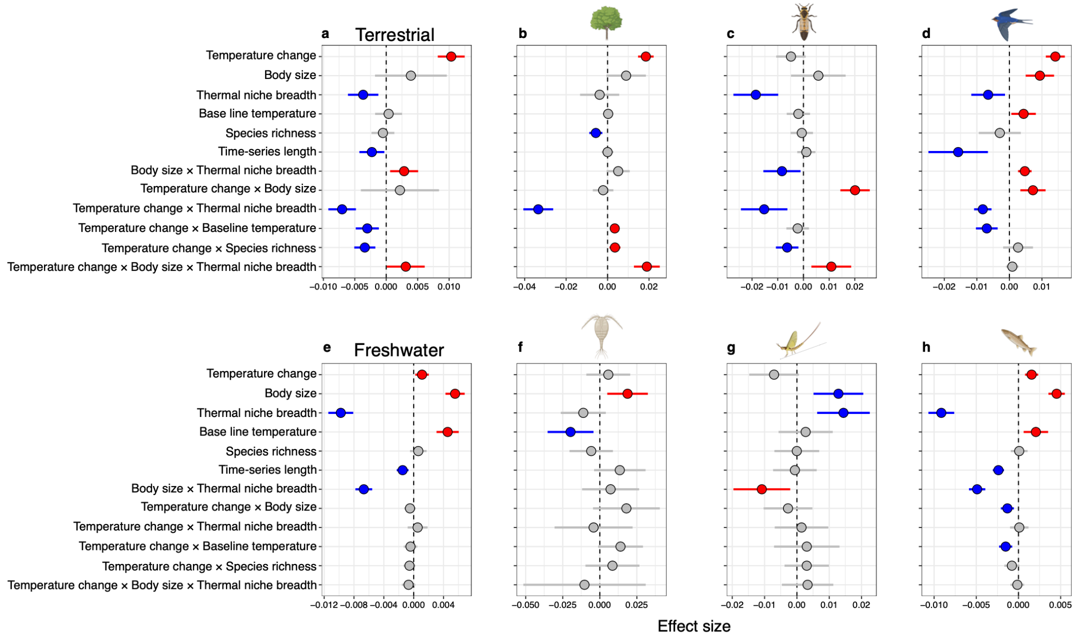

Fig. S4| Predictors of thermophilisation across realms and taxonomic groups (excluding the tropical and polar communities). The plotted points show mean effect sizes of temperature change, mean community body size, mean thermal niche breadth, baseline mean annual temperature, time-series length and species richness on the rates of thermophilisation. Panels **a** and **e** are for all the taxonomic groups plotted for the terrestrial and the freshwater realms, respectively; panels **b-d** and **f-h** are plotted for particular taxonomic groups: plants, terrestrial insects, and birds (**b-d**), and zooplankton, aquatic insects, and fish (**f-h**). Please note that the x-axis range differs among the panels, though the dashed line for zero is the same on all plots. For each realm, effect sizes were calculated after removing outliers that lie beyond two standard deviations from the mean, and after accounting for the effects of taxonomic group (random factor), study id (random factor) and spatial autocorrelation. We also estimated the interaction effects of body size, thermal niche breadth, baseline temperature and temperature change. Effect sizes with grey circles are not significantly different from zero based upon error bars represent 95% confidence intervals are overlapping zero. Silhouettes were created with BioRender.com

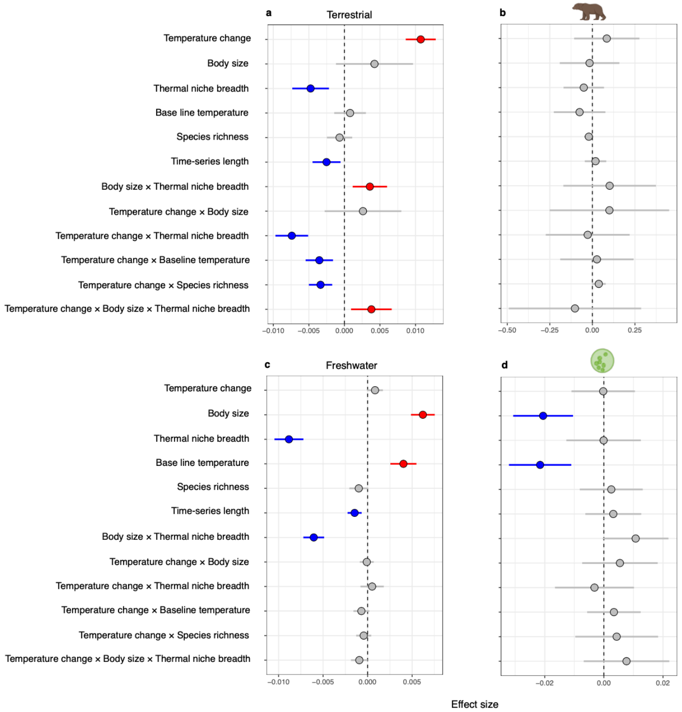

Fig. S5| Predictors of thermophilisation across realms and taxonomic groups (including mammals and phytoplankton). The plotted points show mean effect sizes of temperature change, mean community body size, mean thermal niche breadth, baseline mean annual temperature, time-series length and species richness on the rates of thermophilisation. Panels **a** and **c** are for all the taxonomic groups plotted for the terrestrial and the freshwater realms, respectively; panels **b** and **d** are plotted for mammals and phytoplankton. Please note that the x-axis range differs among the panels, though the dashed line for zero is the same on all plots. For each realm, effect sizes were calculated after removing outliers that lie beyond two standard deviations from the mean, and after accounting for the effects of taxonomic group (random factor), study id (random factor) and spatial autocorrelation. We also estimated the interaction effects of body size, thermal niche breadth, baseline temperature and temperature change. Effect sizes with grey circles are not significantly different from zero based upon error bars represent 95% confidence intervals are overlapping zero. Silhouettes were created with BioRender.com

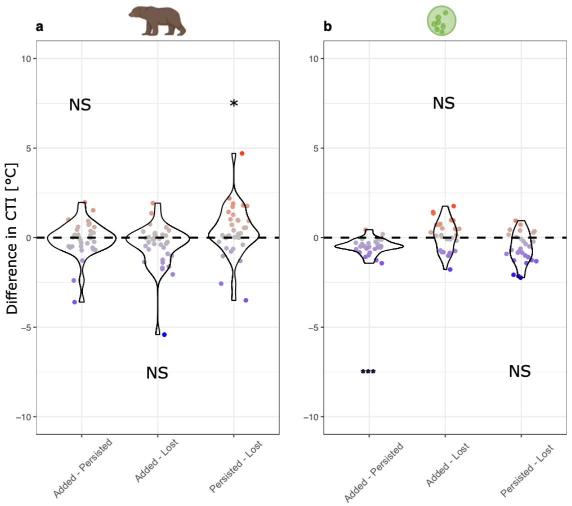

Fig. S6| Contributions of immigration and extirpation to thermophilisation. Difference in the mean thermal affinities of species that immigrated (added), that persisted or that were extirpated (lost) from individual communities. The individual points represent pairwise differences in the mean thermal affinities between these groups (i.e. added, persisted or lost species) for each community. Asterisks indicate whether differences are significantly more frequently above or below zero than expected based on a 0.5 probability (binomial-test); this is indicated by the position of the asterisks above or below zero on the y-axis. * indicates P < 0.05, ** indicates P < 0.005, ***indicates P < 0.001. Silhouettes were created with BioRender.com

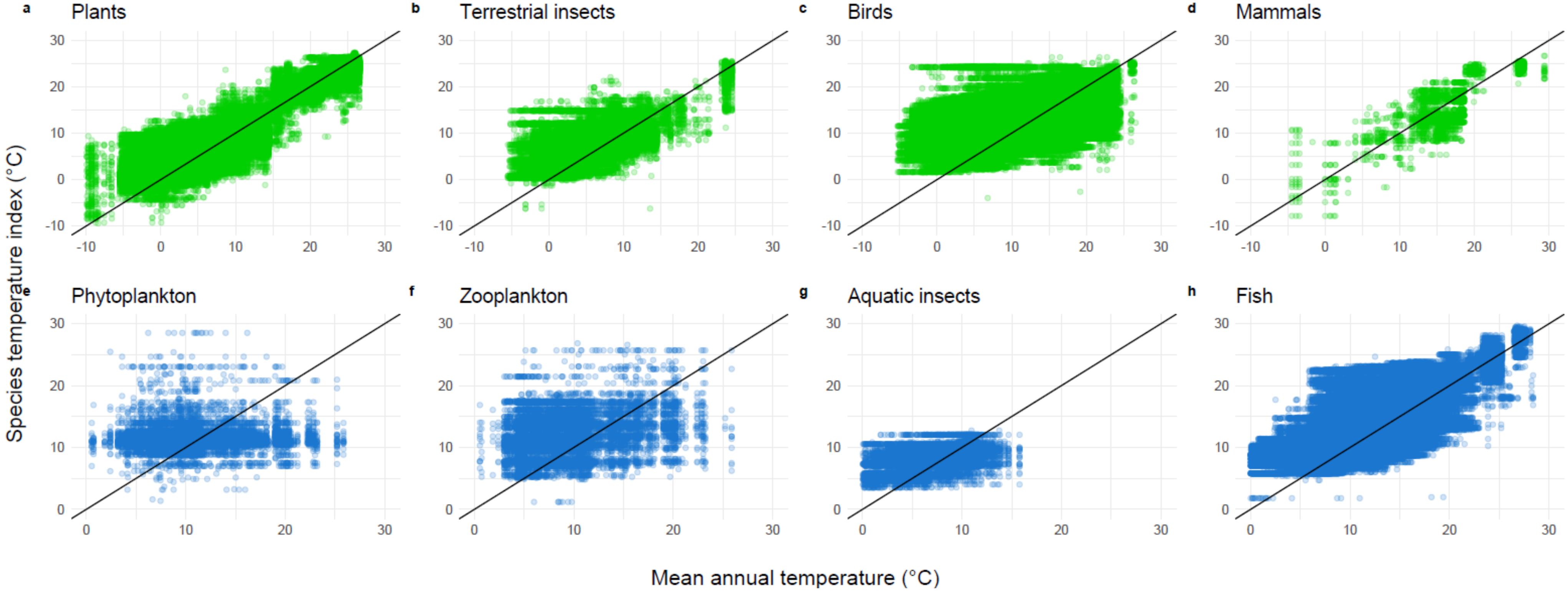

Fig. S7| Species temperature index (STI) and mean annual temperatures for each community. STI was calculated as the average mean annual temperature across each species distributional range. The solid black line indicates a 1:1 relationship between STI and average mean annual temperature. Data points above the black line indicate that STI is higher than the average mean annual temperature at a local site and vice versa.

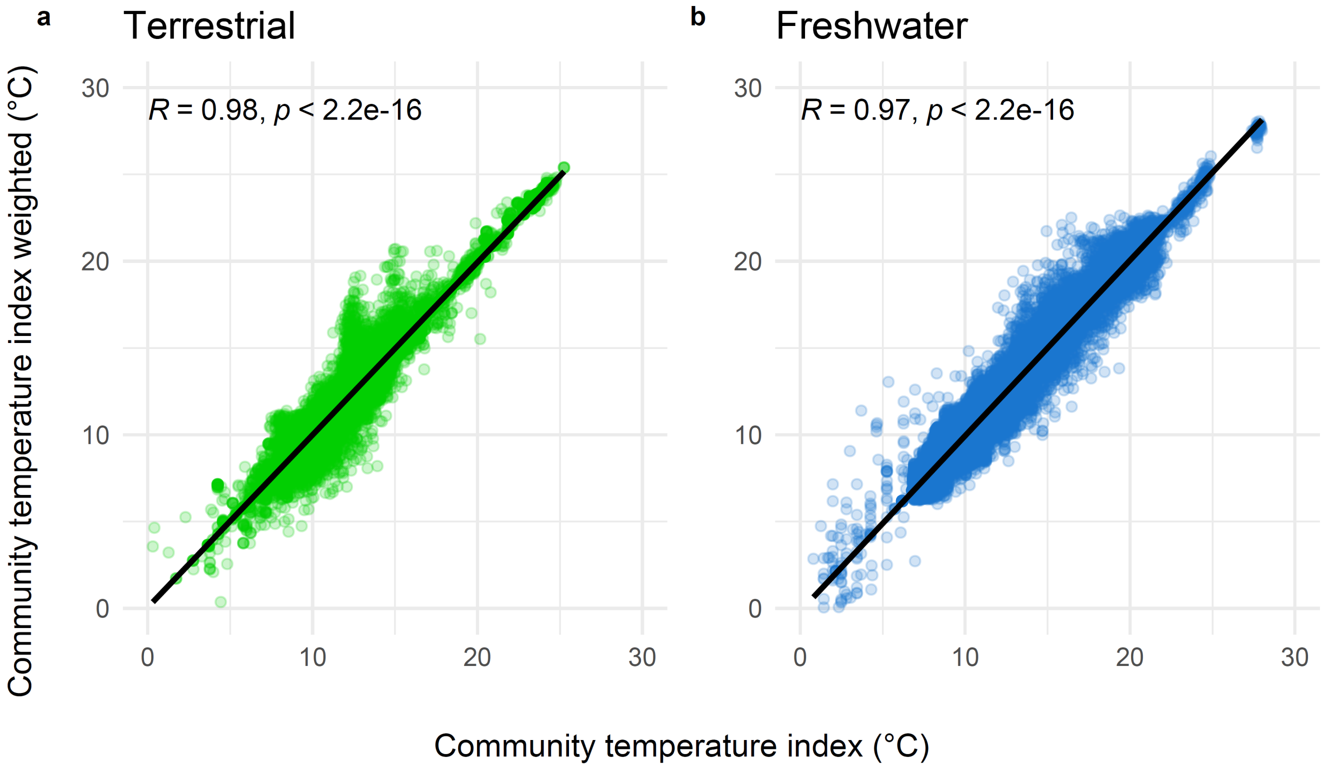

Fig. S8| Relationship between two measures of CTI. Relationship between the community temperature index (CTI) weighted by the abundance of species (y-axis) and the CTI based upon species occurrence data (x-axis) show highly correlated values.

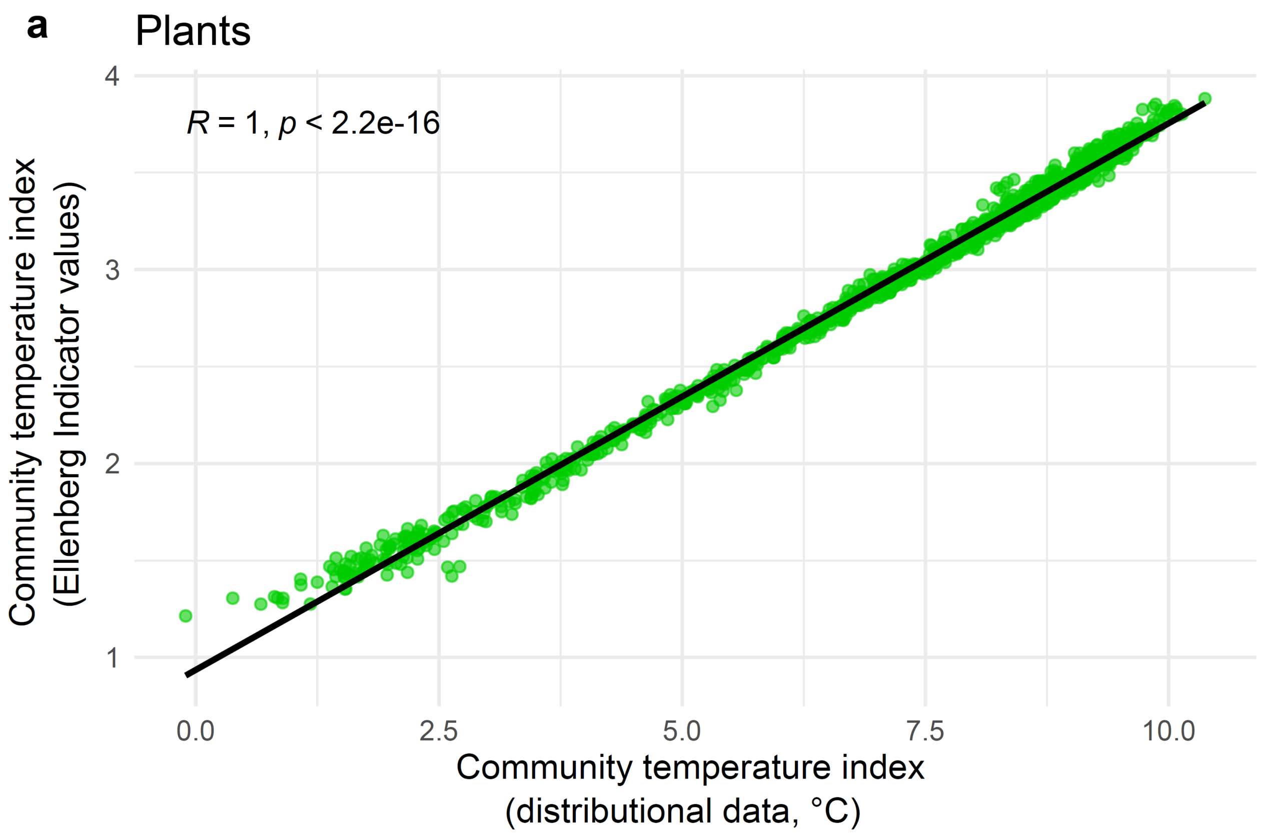

Fig. S9| Plant CTI estimated from distributional data and from Ellenberg's T indicative values. For plants CTI was calculated using species level distributional data downloaded from GBIF and classification taken from the Ellenberg's[49] temperature indicative values. CTI calculated based upon two measures yielded highly correlated values.

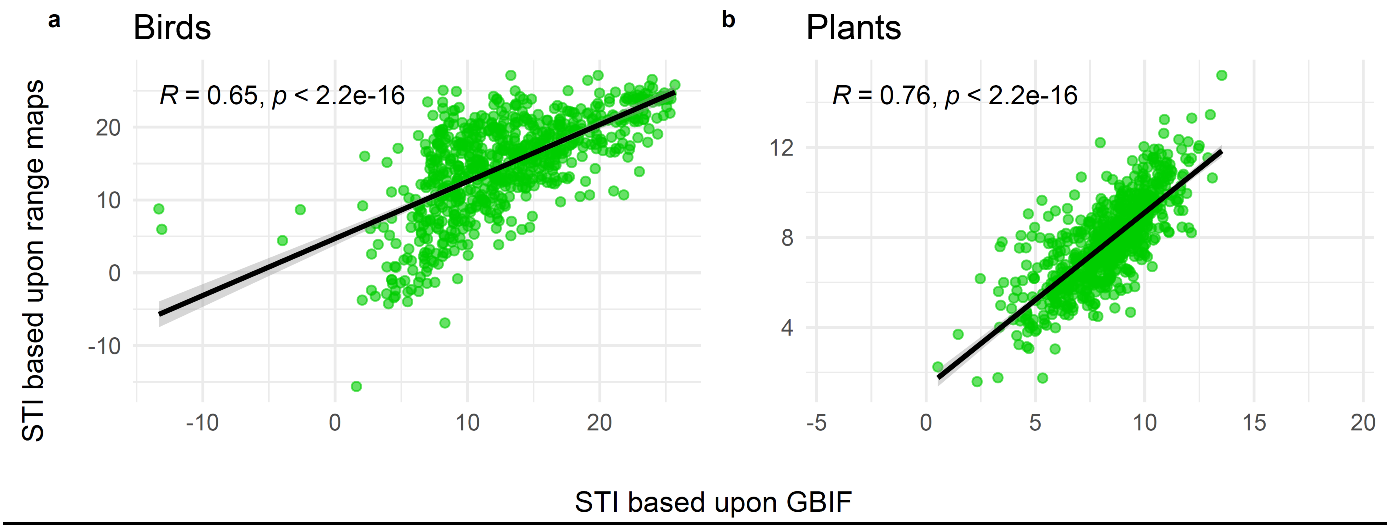

Fig. S10| STIs estimated using shape files of species' ranges against species STIs estimated from GBIF distributional data. We downloaded range maps from the BirdLife dataset for birds and for plants we used the thermal preference data from reference[8] based upon range maps. We overlayed the mean annual temperature layer on the range maps and calculated the species thermal preference for each species as average mean annual temperatures across the whole geographic distribution separately. We also calculated the average mean annual temperature using GBIF data. The correlations indicate that the two measures are comparable and yield highly correlated values.

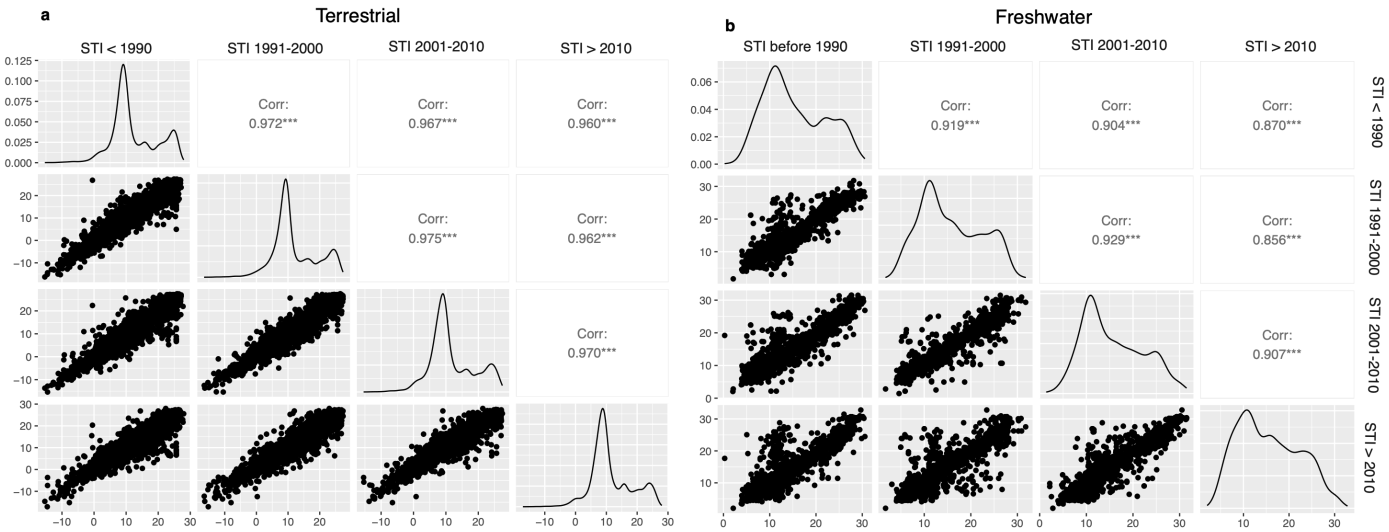

Fig. S11 | STI estimated using distributional data before year Pre-1990, 1990-1991, 2001-2010, and post-2010 periods. For all taxonomic groups, STIs were calculated by sub-setting the GBIF occurrence data using above time bins. STI values for both the realms across all measures are highly correlated. Occurrence records for mammals and phytoplankton are not included.

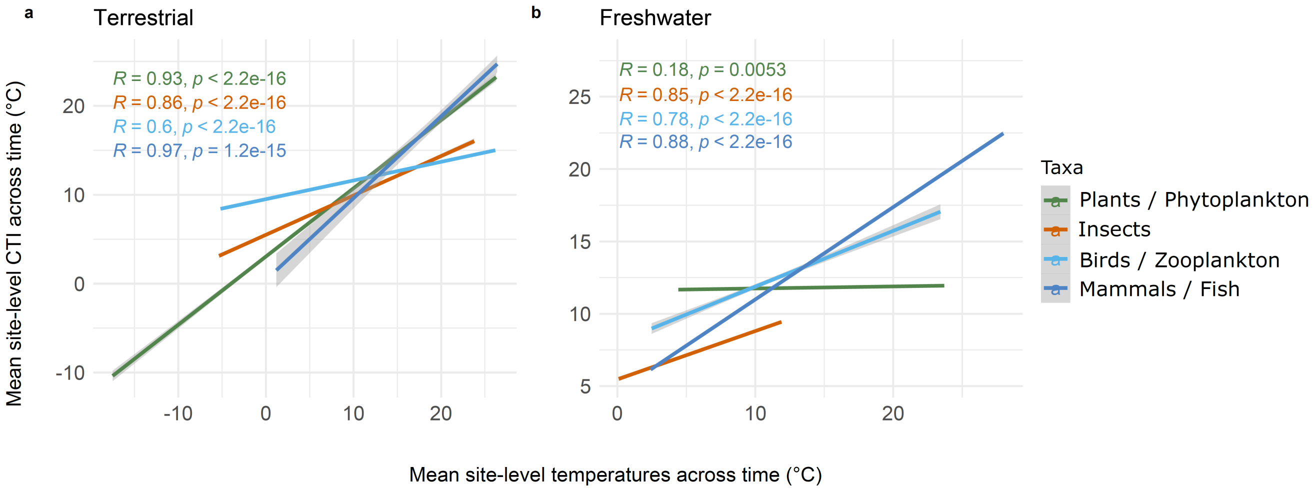

Fig. S12 | CTI plotted as a function of the local sites' mean annual temperature. Site-level mean CTI and site-level mean annual temperatures were calculated across all years for each site. There is a positive relationship observed for all the taxa. All relationships are statistically significant at α=0.05, except for phytoplankton.

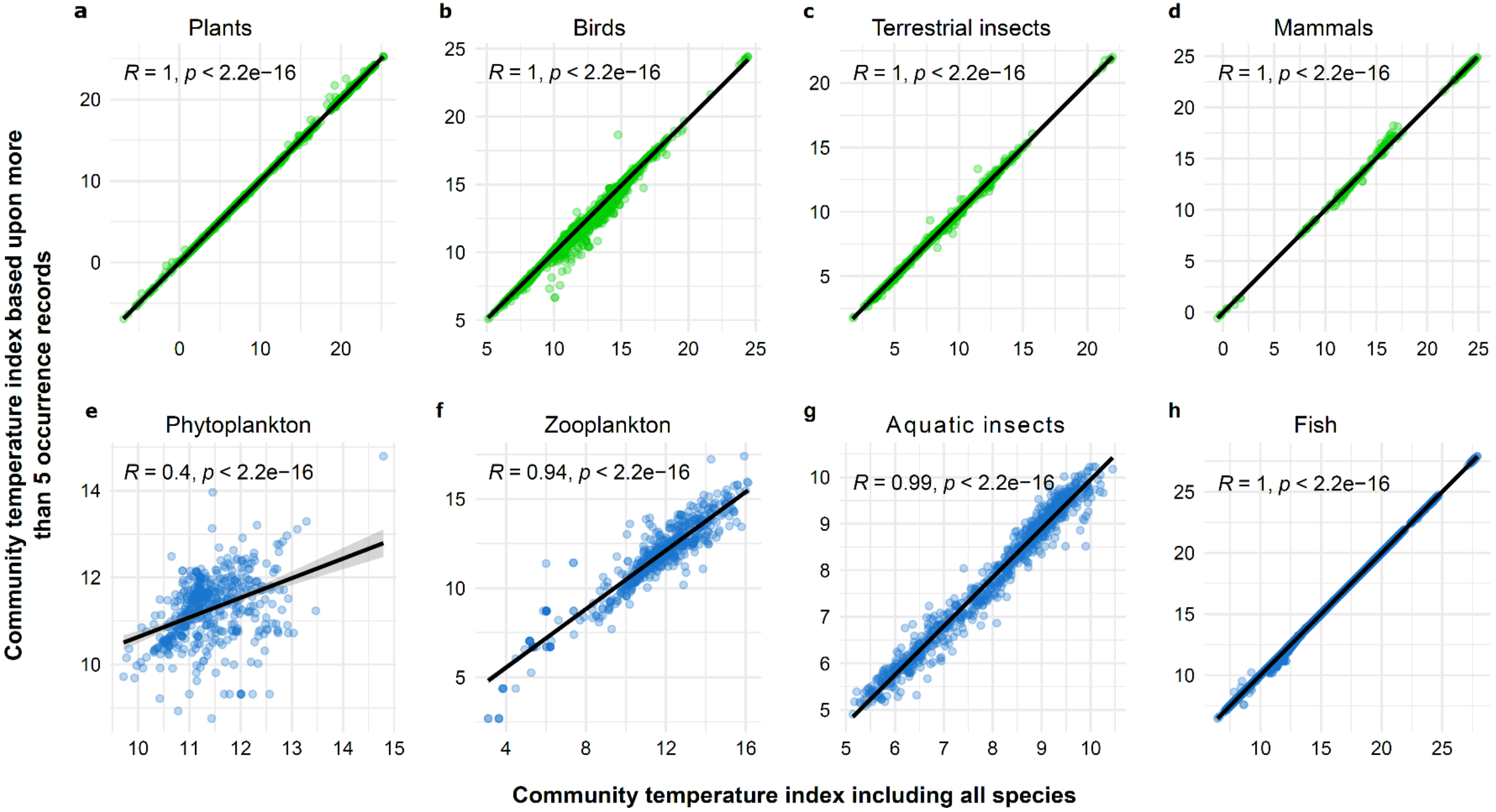

Fig. S13 | Relationship of CTI based upon all the species present in community and CTI based upon species with more than 5 occurrence records. CTI values on y-axis were calculated while excluding all species for which less than five occurrence information was available. On the x-axis, we included all the species present in a community.

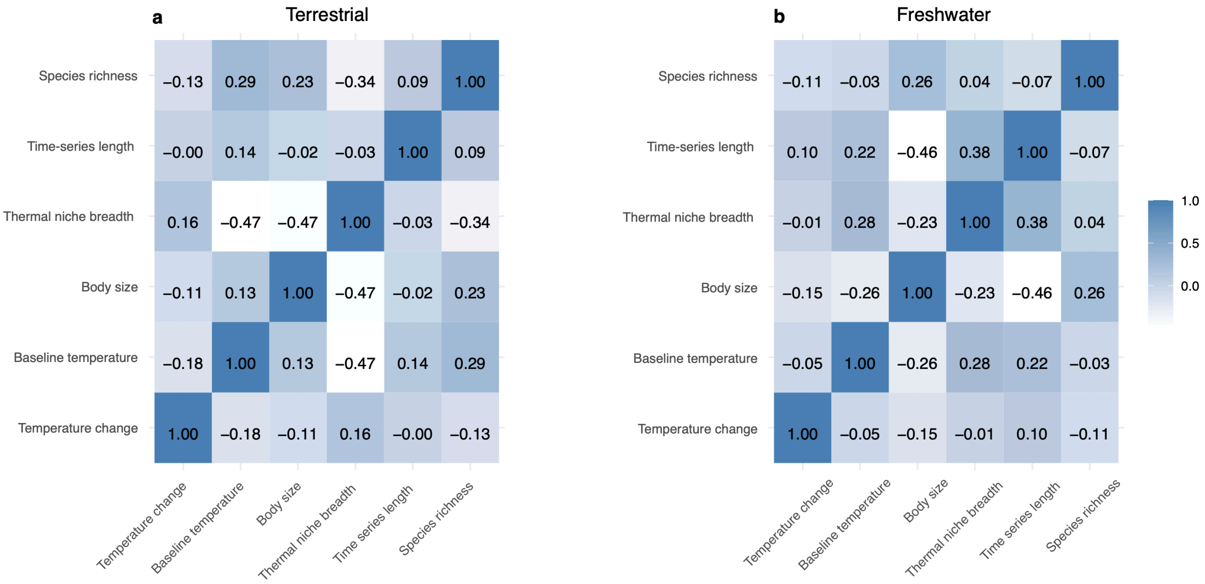

Fig. S14 | Correlation matrices of predictor variables for aquatic and terrestrial realms. Colours range from white (negative correlation) to dark blue (positive correlation).

Supplementary Tables

Supplementary Table S1. Relationships between thermophilisation, temperature change and realm. In a linear mixed effect model (two-sided), thermophilisation was modelled as a function of temperature change and realm (categorical) while accounting for spatial autocorrelation and taxonomic group and study ID as random factors. We added an interaction term between temperature change and realm. Parameter estimates, standard errors and significance levels are given. Bold indicates statistical significance.

Thermophilisation ~ Temperature change * Realm + random = ~1|taxonomic_group /study ID

Predictor	slope	Std.Error	t-value	p-value	R-squared
Temperature change	0.00327293	0.009641461	0.339464	0.7343	0.004
Realm (Terrestrial)	-0.0140162	0.011045932	-1.268902	0.2733	
Temperature change × Realm	**0.04999862**	**0.013961832**	**3.581093**	**0.0003**	

Supplementary Table S2. Relationships between thermophilisation, temperature change, community body size, community thermal niche breadth, baseline temperature, time-series length and species richness for each realm. Thermophilisation was modelled as a function of temperature change, body size, thermal niche breadth, time-series length, and species richness as fixed factors while adding taxonomic group as random factor with study ID nested within taxonomic group. Additionally, we accounted for spatial autocorrelation in a linear mixed model (two-sided). We added interaction terms among all predictor variables. We fitted a model for each realm separately. Parameter estimates, standard errors and significance levels are also given. Bold indicates statistical significance.

Thermophilisation ~ Temperature change * (body size *thermal niche breadth + species richness+ baseline temperature) + Time-series length + random = ~1|taxonomic group / study ID

	Predictor	β	S.E ±	t-value	p-value	R-squared
**Terrestrial (6162)**	**Temperature change**	**0.0107**	**0.001083**	**8.832544**	**<0.001**	**0.027**
	Body size	0.0038	0.0028	1.362	0.1731	
	**Thermal niche**	**-0.004**	**0.0013**	**-3.184**	**0.0015**	
	Baseline temperature	0.0005	0.0011	0.534	0.5930	
	Species richness	-0.0006	0.0009	-0.689	0.4930	
	**Time-series length**	**-0.0023**	**0.0010**	**-2.288**	**0.0221**	
	**Body size × thermal niche**	**0.0032**	**0.0012**	**2.6325**	**0.0085**	
	Temperature change × body size	0.0025	0.0027	0.9013	0.3674	
	**Temperature change × thermal niche**	**-0.0075**	**0.0011**	**-6.4366**	**<0.001**	
	**Temperature change × Baseline temperature**	**-0.0033**	**0.0009**	**-3.4277**	**<0.001**	
	**Temperature change × species richness**	**-0.0034**	**0.0008**	**-4.0510**	**<0.001**	
	**Temperature change × body size × thermal niche**	**0.00370**	**0.0014**	**2.5962**	**0.009**	
**Freshwater (6953)**	**Temperature change**	**0.00108**	**0.00044**	**2.4237**	**0.0154**	**0.091**
	**Body size**	**0.00627**	**0.00065**	**9.5624**	**<0.001**	
	**Thermal niche**	**-0.0095**	**0.00084**	**-11.248**	**<0.001**	

**Baseline temperature**	**0.0043**	**0.00077**	**5.7010**	**<0.001**
Species richness	0.0005	0.00056	1.0562	0.2909
**Time-series length**	**-0.0016**	**0.00039**	**-4.2546**	**<0.001**
**Body size × thermal niche**	**-0.0062**	**0.00058**	**-10.824**	**<0.001**
Temperature change × body size	-0.0004	0.00039	-1.2516	0.2108
Temperature change × thermal niche	0.00117	0.00067	0.1722	0.8632
Temperature change × Baseline temperature	-0.00013	0.00045	-0.2910	0.7710
Temperature change × species richness	-0.00056	0.00039	-1.4222	0.1550
**Temperature change × body size × thermal niche**	**-0.0010**	**0.00046**	**-2.2864**	**0.0223**

278

Supplementary Table S3. Relationships between thermophilisation, temperature change, community body size, community thermal niche breadth, baseline temperature, time-series length and species richness for each taxonomic group. Thermophilisation was modelled as a function of temperature change, body size, thermal niche breadth, time-series length, and species richness as fixed factors while adding study ID as a random factor. Additionally, we accounted for spatial autocorrelation in a linear mixed model (two-sided). We added interaction terms among all predictor variables. Parameter estimates, standard errors and significance levels are also given. The numbers in brackets indicate the number of communities for each taxonomic group after excluding the outliers and communities for which body size data was not available). Bold indicates statistical significance.

Thermophilisation ~ Temperature change * (body size *thermal niche breadth + species richness+ baseline temperature) +Time-series length + random = ~1| study ID

	Predictor	β	S.E ±	t-value	p-value	R-squared
**Plants**	**Temperature change**	**0.0076**	**0.0018**	**4.2412**	**<0.001**	0.26
**(n = 758)**	**Body size**	**0.0083**	**0.0033**	**2.4734**	**0.013**	
	**Thermal niche**	**-0.0089**	**0.0030**	**-2.9337**	**0.003**	
	Baseline temperature	0.00005	0.0012	0.0463	0.963	
	Species richness	-0.0026	0.0014	-1.8088	0.070	
	Time-series length	-0.0001	0.0015	-0.0974	0.9223	
	**Body size × thermal niche**	**0.0119**	**0.0026**	**4.5980**	**<0.001**	
	Temperature change × body size	0.0029	0.0019	1.5133	0.130	
	Temperature change × thermal niche	-0.0052	0.0028	-1.8267	0.068	
	Temperature change × Baseline temperature	0.0018	0.0009	1.8720	0.061	
	Temperature change × species richness	0.0007	0.0012	0.6152	0.538	
	**Temperature change × body size × thermal niche**	**0.0159**	**0.0034**	**4.5792**	**<0.001**	
**Terrestrial insects**	Temperature change	-0.0031	0.0026	-1.1953	0.232	0.19

**(n = 706)**	Body size	0.0041	0.0053	0.7713	0.440	
	**Thermal niche**	**-0.0168**	0.0042	-3.9447	<0.001	
	Baseline temperature	-0.0029	0.0022	-1.3396	0.180	
	Species richness	-0.0007	0.0022	-0.3531	0.724	
	Time-series length	0.0011	0.0018	0.6444	0.519	
	**Body size × thermal niche**	**-0.0067**	0.0034	-1.9549	0.051	
	**Temperature change × body size**	**0.0213**	0.0027	7.8011	<0.001	
	**Temperature change × thermal niche**	**-0.0182**	0.0041	-4.4193	<0.001	
	Temperature change × Baseline temperature	-0.0011	0.0020	-0.5411	0.588	
	**Temperature change × species richness**	**-0.006**	0.0022	-2.7235	0.006	
	Temperature change × body size × thermal niche	0.008	0.0034	2.4575	0.014	
**Birds**	**Temperature change**	0.0140	0.0014	9.451	<0.001	0.082
**(n = 4712)**	**Body size**	0.0093	0.0022	4.221	<0.001	
	Thermal niche	-0.0067	0.0026	-2.581	0.009	
	**Baseline temperature**	0.0045	0.0019	2.351	0.018	
	**Species richness**	-0.0030	0.0032	-0.937	0.348	
	**Time-series length**	-0.0146	0.0045	-3.184	0.001	
	**Body size × thermal niche**	0.0047	0.0010	4.497	<0.001	
	**Temperature change × body size**	0.0072	0.0019	3.654	<0.001	
	Temperature change × thermal niche	-0.0081	0.0013	-5.965	<0.001	
	**Temperature change × Baseline temperature**	-0.0069	0.0017	-4.072	<0.001	
	**Temperature change × species richness**	0.0025	0.0023	1.113	0.265	
	Temperature change × body size × thermal niche	0.0009	0.0009	0.920	0.357	
**Mammals**	Temperature change	0.084	0.097	0.865		0.4
**(n = 23)**	Body size	-0.016	0.089	-0.184		
	Thermal niche	-0.050	0.060	-0.832		
	Baseline temperature	-0.075	0.077	-0.973		

	Species richness	-0.020	0.013	-1.509		
	Time-series length	0.0185	0.032	0.578		
	Body size × thermal niche	0.101	0.138	0.731		
	Temperature change × body size	0.100	0.178	0.558		
	Temperature change × thermal niche	-0.027	0.125	-0.218		
	Temperature change × Baseline temperature	0.026	0.109	0.242		
	Temperature change × species richness	0.0377	0.020	1.833		
	Temperature change × body size × thermal niche	-0.102	0.198	-0.515		
**Phytoplankton (n = 144)**	Temperature change	-0.0002	0.0054	-0.0410	0.9673	0.29
	**Body size**	-0.0205	0.0051	-3.9808	<0.001	
	Thermal niche	-0.00008	0.0064	-0.0131	0.9895	
	**Baseline temperature**	-0.0215	0.0053	-4.0094	<0.001	
	Species richness	0.00250	0.0054	0.4609	0.6456	
	Time-series length	0.00318	0.0048	0.6601	0.6285	
	**Body size × thermal niche**	0.0107	0.0056	1.9001	0.0597	
	Temperature change × body size	0.0054	0.0065	0.8336	0.4060	
	Temperature change × thermal niche	-0.0031	0.0068	-0.4662	0.6418	
	Temperature change × Baseline temperature	0.0033	0.0046	0.7350	0.4636	
	**Temperature change × species richness**	0.0043	0.0071	0.6066	0.5451	
	Temperature change × body size × thermal niche	0.0076	0.0073	1.0382	0.3011	
**Zooplankton (n = 222)**	Temperature change	0.006	0.008	0.695	0.487	0.16
	Body size	0.005	0.007	0.743	0.458	
	**Thermal niche**	**-0.021**	**0.009**	**-2.202**	**0.028**	
	Baseline temperature	-0.015	0.008	-1.883	0.061	
	Species richness	-0.006	0.007	-0.940	0.348	
	Time-series length	0.011	0.007	1.567	0.118	
	Body size × thermal niche	0.026	0.014	1.909	0.057	
	Temperature change	0.018	0.013	1.370	0.172	

	× body size					
	Temperature change × thermal niche	-0.012	0.014	-0.897	0.370	
	Temperature change × Baseline temperature	0.009	0.007	1.183	0.237	
	Temperature change × species richness	0.014	0.008	1.603	0.110	
	Temperature change × body size × thermal niche	0.019	0.024	0.781	0.435	
**Aquatic insects (n = 422)**	Temperature change	-0.007	0.003	-1.805	0.071	0.06
	**Body size**	**0.012**	**0.003**	**3.268**	**0.001**	
	**Thermal niche**	**0.014**	**0.004**	**3.466**	**0.0005**	
	Baseline temperature	0.002	0.004	0.632	0.527	
	Species richness	-0.00003	0.003	-0.010	0.991	
	Time-series length	-0.0006	0.003	-0.181	0.856	
	**Body size × thermal niche**	**-0.010**	**0.004**	**-2.420**	**0.015**	
	Temperature change × body size	-0.002	0.003	-0.717	0.473	
	Temperature change × thermal niche	0.001	0.004	0.338	0.735	
	Temperature change × Baseline temperature	0.003	0.005	0.590	0.555	
	Temperature change × species richness	0.003	0.003	0.868	0.385	
	Temperature change × body size × thermal niche	0.003	0.004	0.813	0.416	
**Fish (n = 6207)**	**Temperature change**	**0.0017**	**0.0004**	**4.3518**	**<0.001**	0.10
	**Body size**	**0.0046**	**0.0005**	**9.2420**	**<0.001**	
	**Thermal niche**	**-0.0088**	**0.0008**	**-11.6007**	**<0.001**	
	**Baseline temperature**	**0.0025**	**0.0008**	**3.3238**	**<0.001**	
	Species richness	-0.0002	0.0005	-0.4164	0.6771	
	**Time-series length**	**-0.0023**	**0.0003**	**-6.7985**	**<0.001**	
	**Body size × thermal niche**	**-0.0048**	**0.0005**	**-9.5208**	**<0.001**	
	**Temperature change × body size**	**-0.0014**	**0.0004**	**-3.5237**	**<0.001**	
	Temperature change × thermal niche	0.0000	0.0006	0.0849	0.9323	
	**Temperature change × Baseline temperature**	**-0.0017**	**0.0004**	**-3.9748**	**<0.001**	

**Temperature change × species richness**	-0.0004	0.0005	-0.9656	0.3343
Temperature change × body size × thermal niche	0.0002	0.0004	0.4463	0.6554

Supplementary Table S4. Table showing the mean of number of individuals sampled for each of the taxonomic group (for the communities for which we have the abundance information available). We excluded plants as abundance information for plants was not always as count data.

Taxonomic group	Mean number of individuals sampled
Birds	212
Terrestrial insects	89
Mammals	44
Fish	223
Phytoplankton	465
Zooplankton	709
