# Supplementary material for: Warming underpins community turnover in temperate freshwater and terrestrial communities
Source: Nat Commun. 2024 Mar 1;15:1921. doi: 10.1038/s41467-024-46282-z (PMC10907361; doi:10.1038/s41467-024-46282-z)
Supplement: Supplementary file 3 — Reporting Summary [file 41467_2024_46282_MOESM3_ESM.pdf]

nature portfolio

Corresponding author(s): Imran Khaliq, Anita Narani

Last updated by author(s): 24/01/2024

Reporting Summary

Nature Portfolio wishes to improve the reproducibility of the work that we publish. This form provides structure for consistency and transparency in reporting. For further information on Nature Portfolio policies, see our Editorial Policies and the Editorial Policy Checklist.

Statistics

For all statistical analyses, confirm that the following items are present in the figure legend, table legend, main text, or Methods section.

n/a	Confirmed	
☐	☒	The exact sample size (*n*) for each experimental group/condition, given as a discrete number and unit of measurement
☒	☐	A statement on whether measurements were taken from distinct samples or whether the same sample was measured repeatedly
☐	☒	The statistical test(s) used AND whether they are one- or two-sided<br>*Only common tests should be described solely by name; describe more complex techniques in the Methods section.*
☐	☒	A description of all covariates tested
☐	☒	A description of any assumptions or corrections, such as tests of normality and adjustment for multiple comparisons
☐	☒	A full description of the statistical parameters including central tendency (e.g. means) or other basic estimates (e.g. regression coefficient) AND variation (e.g. standard deviation) or associated estimates of uncertainty (e.g. confidence intervals)
☒	☐	For null hypothesis testing, the test statistic (e.g. *F*, *t*, *r*) with confidence intervals, effect sizes, degrees of freedom and *P* value noted<br>*Give P values as exact values whenever suitable.*
☒	☐	For Bayesian analysis, information on the choice of priors and Markov chain Monte Carlo settings
☒	☐	For hierarchical and complex designs, identification of the appropriate level for tests and full reporting of outcomes
☐	☒	Estimates of effect sizes (e.g. Cohen's *d*, Pearson's *r*), indicating how they were calculated

Our web collection on statistics for biologists contains articles on many of the points above.

Software and code

Policy information about availability of computer code

Data collection	We used publically available time-series data of ecological communities compiled from several sources: BioTime (https://biotime.st-andrews.ac.uk/aboutUs.php), National Lake Assessment (US Environmental Protection Agency, National Lakes Assessment Surveys, https://www.epa.gov/national-aquatic-resource-surveys/nla), RivFishTime (http://dx.doi.org/10.1111/geb.13210), several Swiss terrestrial insect studies and Biodiversity Monitoring Switzerland (BDM,). BDM data can be accessed upon request from BDM (https://www.biodiversitymonitoring.ch/index.php/en/home-footer). For occurrences records of species, we downloaded data from Global Biodiversity Information Facility (GBIF,https://www.gbif.org/). Temperature data was downloaded from Chelsa dataset (https://chelsa-climate.org/). Body size data for the species was compiled from several sources including Fishbase (https://www.fishbase.se/search.php), TRY plant database (https://www.try-db.org/TryWeb/Home.php), for birds and mammals from Etard et al. 2020. For the remaining taxa including insects, phytoplankton and zooplankton, we compiled data directly from the primary literature (list of sources will be provided along with the data upon request). Data and R codes to reproduce the results are available at Figshare: 10.6048/m9.figshare.25052696
Data analysis	We analysed all the data in R version 4.1.2.

For manuscripts utilizing custom algorithms or software that are central to the research but not yet described in published literature, software must be made available to editors and reviewers. We strongly encourage code deposition in a community repository (e.g. GitHub). See the Nature Portfolio guidelines for submitting code & software for further information.

March 2021

Data

Policy information about availability of data

All manuscripts must include a data availability statement. This statement should provide the following information, where applicable:

- Accession codes, unique identifiers, or web links for publicly available datasets
- A description of any restrictions on data availability
- For clinical datasets or third party data, please ensure that the statement adheres to our policy

Part of the data that support the findings of this study are available from Biodiversity Monitoring Switzerland (BDM) but restrictions apply to the availability of these data, which were used under license for the current study, and so are not publically available. The rest of the data along with R-codes will be publisjed online upon acceptance of the paper. Species level body size data are however available from the authors upon request.

Field-specific reporting

Please select the one below that is the best fit for your research. If you are not sure, read the appropriate sections before making your selection.

☐ Life sciences ☐ Behavioural & social sciences ☒ Ecological, evolutionary & environmental sciences

For a reference copy of the document with all sections, see nature.com/documents/nr-reporting-summary-flat.pdf

Ecological, evolutionary & environmental sciences study design

All studies must disclose on these points even when the disclosure is negative.

Study description	We estimated the rate of warming-related community turnover termed as thermophilisation of freshwater and terrestrial communities using 13,324 community-level time-series of duration between five and 38 years and representing eight major taxonomic groups. We further explored the relative effects of biotic and abiotic predictors, including the rate of warming, mean community body size, niche breadth baseline temperature and species richness. We conclude that warming is the major driver of community turn-over in both the freshwater and terrestrial realms.
Research sample	We used publically available time-series data of ecological communities compiled from several sources: BioTime, National Lake Assessment (US Environmental Protection Agency, National Lakes Assessment Surveys, RivFishTime, several Swiss terrestrial insect studies and Biodiversity Monitoring Switzerland (BDM). BDM data can be accessed upon request from BDM. For occurrences records of species, we downloaded data from Global Biodiversity Information Facility (GBIF). Temperature data was downloaded from Chelsa datasets. Body siye data for species was compiled from several sources including Fishbase, TRY plant database, for birds and mammals from Etard et al. 2020. For the remaining taxa including insects, phytoplankton and zooplankton, we compiled data directly from the primary literature (list of sources will be provided along with the data upon request).
Sampling strategy	We did not apply any statistical method to predetermine the sample size. We used large time-series data of 13302 communities of eight major taxonomic groups.
Data collection	We used publicly available time-series data of ecological communities. The information about species presence and absence was systematically recorded at each unique community and were aggregated at year level. These data were compiled from several studies that was collected by hundreds of researchers over the years spread across the globe. These data were collected and standardised by the data providers.
Timing and spatial scale	We used time-series data of ecological communities of eight major taxonomic groups sampled between 1980-2019 across the globe. The data is aggregated at year level. The duration of time-series varies from five to 38 years for different taxonomic groups.
Data exclusions	In the analysis of quantifying the influence of drivers on thermophilisation, to meet the assumption of the normality of residuals, we excluded all data points as outliers that were beyond 2 standard deviations from the mean of all the residuals of each model.
Reproducibility	We did not perform any experiment and used already available data.
Randomization	To quantify the effects of biotic and abiotic drivers on thermophilisation at realm level, we accounted for the effects of taxonomic groups on thermophilisation by adding taxonomic groups as a random factor.
Blinding	For our study blinding is not relevant as we looked at warming-related changes in ecological communities of freshwater and terrestrial realms.

Did the study involve field work? ☐ Yes ☒ No

Reporting for specific materials, systems and methods

We require information from authors about some types of materials, experimental systems and methods used in many studies. Here, indicate whether each material, system or method listed is relevant to your study. If you are not sure if a list item applies to your research, read the appropriate section before selecting a response.

Materials & experimental systems

n/a	Involved in the study
☒	☐ Antibodies
☒	☐ Eukaryotic cell lines
☒	☐ Palaeontology and archaeology
☒	☐ Animals and other organisms
☒	☐ Human research participants
☒	☐ Clinical data
☒	☐ Dual use research of concern

Methods

n/a	Involved in the study
☒	☐ ChIP-seq
☒	☐ Flow cytometry
☒	☐ MRI-based neuroimaging
